# Supplementary material for: Are carbon emissions trading and green financial instruments synergistic? -Comprehensive quantitative research based on content analysis
Source: PLoS One. 2024 Mar 7;19(3):e0298601. doi: 10.1371/journal.pone.0298601 (PMC10919601; doi:10.1371/journal.pone.0298601)
Supplement: S1 Data — (ZIP) [file pone.0298601.s001.zip › Data/地级市Z_F工作报告.zip!/╔╜╬≈/│ñ╓╬╩╨/└┤╫╘╣1⁄2╓┌║┼í╛┬φ┐╦╩2╛▌═°í┐_2012.docx]

各位代表：

现在，我代表市人民政府向大会作工作报告，请予审议，并请市政协各位委员和其他列席人员提出意见。

一、过去五年工作回顾

本届政府是2007年市十二届人大第一次会议选举产生的，现任期已满。五年来，在省委、省政府和市委的正确领导下，在市人大、市政协的监督帮助下，我们坚持以科学发展观为指导，抢抓机遇、创新举措，齐心协力、狠抓落实，大规模投资、大创新发展、大力度惠民，圆满完成了市十二届人大一次会议确定的五年目标任务，经济社会步入快速发展的新阶段，为建设全国最宜居最宜发展城市奠定了坚实基础。

（一）经济发展有大跨越。 2011年全市地区生产总值达到1218.6亿元，是2006年的2.6倍，成为全省第二个进入“千亿元俱乐部”的城市。固定资产投资完成686.9亿元，是2006年的3.7倍。财政总收入完成255.5亿元，是2006年的2.5倍；一般预算收入完成104.4亿元，是2006年的2.3倍，是全省第二个突破百亿元大关的城市。城镇居民人均可支配收入达到20131元，是2006年的2倍；农民人均纯收入达到7092元，是2006年的1.8倍。

（二）产业素质有大提高。第一产业特色鲜明，以壮大龙头企业、建设产业化项目、发展设施农业为重点，培育了15家销售收入超亿元的骨干龙头企业，农业产业化龙头企业达到289个，2011年实现销售收入95亿元，农产品加工转化率达到50%。形成了400个“一村一品”专业村、4个省级“一县一业”示范基地，带动从业农户10万户。建成37个国家级农业标准化示范区和95个农业科技园区，成为全国第一个农业综合标准化示范市，初步形成优质玉米、小杂粮、设施蔬菜、食用菌、干鲜果、规模养殖六大特色板块。新农村建设连续四年受到省委、省政府表彰。第二产业亮点纷呈，大力改造提升传统产业，五年共实施重点技改项目491项，完成投资900亿元，是上一个五年的3.8倍。传统产业素质得到提升，形成了资源循环利用和废水、废气、废渣、余热余压高效综合利用的循环发展模式。完成了煤炭资源整合煤矿兼并重组，彻底告别了“小煤窑”，实现了建设大矿井的目标。加快发展新兴产业，五年实施新兴产业项目290个，完成投资470亿元。新建了20个销售收入百亿元以上的非煤非电项目，全国第一桶具有完全自主知识产权的煤基合成油成功出油，全球第一条100万伏特高压输变电线路并网运行，全国唯一一条垂直整合LED全产业链正式贯通。培育了潞安煤基合成油、潞安太阳能、高科华上光电、成功汽车、振东药业、康宝制药、上泓HF燃油乳化、华农纳米碳增效肥等一批高新技术企业，形成了低污染、低排放、高效益的战略性新兴产业集群。 2011年新兴产业增加值108亿元，是2006年的3.3倍。第三产业快速发展，2011年社会消费品零售总额达到321亿元，是2006年的2.4倍。浦发银行、华夏银行、交通银行落户我市，组建了长治农商银行、长子县村镇银行及31个小额贷款公司。开工建设了长治物流中心、太行山农产品物流园区，被商务部确定为现代物流示范市。规划建设了长治文化创意传媒园区，成功举办了中国·长治国际攀岩节、八路军文化节等一批节庆活动，文化产业增加值占地区生产总值的比重达到4.2%。太行山大峡谷、八路军纪念馆等5个景区景点跻身4A级行列，荣获了中国优秀旅游城市、中国旅游品牌十大目的地称号，2011年全市旅游总收入达到126.2亿元，是2006年的3.5倍。

（三）城乡面貌有大变化。按照“一核双圈”的城镇化战略构架，制定了推进市域城镇化实施方案和上党城镇群建设行动计划，上党城镇群基础设施、产业布局和生态建设初步形成体系。 2011年城镇化率达到47.66%，比2006年提高9.66个百分点。建成了客运东站、体育中心、六府塔、长北污水处理厂等一批城建工程。市区垃圾无害化处理率由59%提高到100%，污水处理率由72.9%提高到92.2%，集中供热率由44%提高到86%。深入实施“蓝天碧水”、“两地”保护和浊漳河流域综合治理工程，二氧化硫和化学需氧量排放量分别比2006年下降14.3%和12.1%，超额完成“十一五”减排任务，荣获全省蓝天碧水工程优秀奖。万元生产总值能耗由2006年的3.28吨标煤下降到2011年的2.4吨标煤，下降26.8%。全面推进造林绿化，五年累计造林219万亩，在我市召开了全国造林绿化现场会，荣获国家园林城市、全国绿化模范城市和全省造林绿化突出贡献奖。2011年市区空气质量二级以上天数达到357天，比2006年增加91天，在国家环保部重点监测的113个城市中排名由67位上升到16位，连续8年成为全省空气质量最好的城市，连续4年名列华北第一。

（四）改革开放有大成效。新一轮市县政府机构改革按期完成。事业单位分类改革、医药卫生体制改革进展顺利。集体林权制度改革成效明显，922万亩林地全部确权到户。文化体制改革、财税体制改革、行政审批制度改革进一步深化。国有企业改革步伐加快，改制面达到66.7%，长钢与首钢成功重组，实现了我市钢铁产业发展的历史性突破。全市民营经济增加值占地区生产总值比重由2006年的52%提高到56.5%。全方位扩大对外开放，2011年进出口总额达到7.8亿美元，是2006年的6.7倍。积极参加中博会、能博会等大型招商活动，引进了荷兰飞利浦、德国西门子等世界500强企业和中国五矿、江苏悦达、红星美凯龙等一批知名企业。振东制药在创业板成功上市。与德国北威州玛尔市等国际友好城市的经济合作进一步加强，与沿海发达地区、环渤海经济圈、中原经济协作区的合作交流更加广泛。

（五）民生质量有大改善。累计投入120多亿元，高标准实施了省里确定的两轮“五个全覆盖”，同步实施了我市的“五个全覆盖”。五年累计新增城镇就业18.8万人，城镇登记失业率控制在3%以内。城镇养老、医疗、失业等社会保险覆盖率达到95%以上，在全省率先实现了全民医保，城乡低保对象应保尽保。开工建设各类保障性住房352.3万平方米、4.38万套，竣工188万平方米、2.32万套，完成农村危房改造8650户。科技创新能力不断增强，荣获国家级科技奖5项，专利年申请量由2006年的92项提高到2011年的1197项，连续七次荣获全国科技进步先进市，被国家科技部等17部委确定为国家可持续发展实验区。人口计生工作成效显著，人口自然增长率控制在6‰以内。双拥共建、国防动员、民兵预备役等工作取得新成绩，荣获全国双拥模范城“四连冠”。认真做好民族宗教工作，被国务院表彰为全国民族团结进步模范集体。新闻出版、体育健身、老龄、妇女儿童、残疾人事业健康发展，文物保护、外事侨务、人民防空、防震减灾、气象、统计、档案、保密、地方志、红十字会、慈善等各项事业全面进步。

（六）安全生产有大创新。把安全生产作为一切工作的底线和第一保障，全力做好本质安全型城市创建工作。认真落实省政府出台的12个行业118条安全生产规定，深入开展安全生产专项整治。实施了安全专项督查、安全联合执法、重大隐患事前责任追究、重大安全隐患挂牌督办、矿级领导带班下井和“5173”等一系列行之有效的安全措施。全面落实政府监管责任和企业主体责任，进一步夯实安全生产基层基础工作。荣获全国安全生产创新一等奖。全市安全生产事故起数和死亡人数分别比2006年下降46%和40.1%，煤矿百万吨死亡率由2006年的0.76下降到0.032，安全生产形势明显好转。

（七）民主法制有大推进。自觉接受人大、政协的监督，认真执行市人大及其常委会的各项决议，支持各民主党派、工商联、无党派人士和人民团体参政议政。人大代表建议和政协委员提案办复率达到100%。积极推进平安长治建设，不断加强社会管理和维稳工作，切实强化应急管理。深入开展法律宣传服务和人民调解，解决了一大批信访突出问题。圆满完成北京奥运会、建国60周年等大型活动安保任务，被中央综治委表彰为全国社会治安综合治理优秀市。

万物皆有时，时来不可失。刚刚过去的2011年，我们紧紧抓住转型综改试验区建设这个大机遇，强力推进工业新型化、农业现代化、市域城镇化和城乡生态化，是各项工作成效最明显、城乡面貌变化最大的一年。项目建设的力度前所未有，全年开工建设重点项目1324项，竣工707项，完成投资970亿元，投资额名列全省第一，一年投资相当于前两年的总和，创造了项目开工数、竣工数和投资完成额三个“历史之最”。招商引资的效果前所未有，全市招商引资签约项目349个，总投资4275亿元，实际到位资金397.2亿元，是前四年到位资金的2倍。其中第六届中博会签约项目241个，名列全省第一，签约项目金额3124亿元，比前五届中博会签约总量的3倍还多。市域城镇化的速度前所未有，开工建设了上党城镇群路网工程和市区“九纵九横”道路畅通、“三河一渠”环城生态水系综合治理等一批重点工程，启动了47个城中村改造，一年新增城镇人口20.8万，城镇化率提高5.88个百分点。改革创新的活力前所未有，在资源配置、项目审批、投资融资、科技人才等方面大胆创新、先行先试，与省国土厅签订合作协议，率先开展城乡建设用地增减挂钩、矿业存量土地整合利用，有效保障了项目用地；建立了12个院士、博士工作站，引进80多名海内外高层次人才；成立了“四化”建设领导组，创新“六七八”推进工作落实机制，实行“三分之一”工作制度，确保了各项工作的有序、高效运行。加快发展的势头前所未有，全市地区生产总值、项目投资双双突破千亿元大关，进入了快速发展的“双千亿”阶段。各项工作全面协调推进，共获得省厅级以上表彰奖励592项，其中国家级37项、省部级241项，特别是一举夺得全国文明城市这一桂冠，成为全省唯一获此殊荣的城市，极大提升了长治的知名度、美誉度和竞争力。在全省年度目标责任综合考评中名列第一，实现了“十二五”良好开局！

各位代表，本届政府所取得的成绩，是省委、省政府正确领导、亲切关怀的结果，是市委统揽全局、科学决策的结果，是市人大、市政协有效监督、大力支持的结果，是全市广大干部群众群策群力、拼搏进取的结果。在此，我代表市人民政府，向全市人民，向驻市部队、武警官兵、公安干警，向各民主党派、人民团体，向尽心履职的人大代表、政协委员以及所有关心支持长治发展的社会各界朋友，表示崇高的敬意和衷心的感谢！

在肯定成绩的同时，我们也清醒地认识到，全市经济社会发展中还存在不少问题：一是当前国内外经济形势错综复杂，经济发展的下行压力加大，不少企业生产经营困难。二是县域经济发展不协调，农业产业化水平不高，农民收入增长还不够快。三是第二产业中传统产业的比重偏大，新兴产业的培育和成长还不够快，服务业和文化产业发展水平低。四是城市建设步伐不快，统筹城乡发展的机制不完善，土地、环境、资金供需矛盾突出。五是关系群众切身利益的就业、教育、医疗、住房、交通等领域还存在不少问题，保障和改善民生需要进一步加大力度。六是一些干部作风涣散、以权谋私，酗酒、赌博、敛财，消极腐败，一些部门政令不畅、执行力不强。对此，我们将采取更加有效的措施，认真加以解决。

二、今后五年目标任务

各位代表，今后五年，我市经济社会发展的主要奋斗目标是：

（一）经济实力再上新台阶，跃入中部城市20强。主要经济指标在现有基础上再翻一番，地区生产总值年均增长15%左右，到2016年达到2700亿元以上，人均生产总值超过1.2万美元，在中部六省88个地级市的排名由现在的前30位进入20强。固定资产投资年均增长25%以上，力争五年累计完成投资1万亿元。财政总收入年均增长20%，2016年达到500亿元以上。城镇居民人均可支配收入年均增长15%，达到4万元左右；农民人均纯收入年均增长15%，达到1.5万元左右，为实现全面小康社会奠定坚实基础。

（二）转型发展形成新格局，构建长治现代产业体系。进一步提高农业产业化水平，农产品加工转化率达到65%以上。加快传统产业改造提升步伐，传统产业循环率达到45%以上。培育壮大光电子、太阳能光伏、新材料、先进装备制造、中西制药、节能环保等新兴产业，新兴产业增加值占工业增加值的比重达到50%以上。快速提升文化旅游、现代物流、金融保险等现代服务业，大力发展生产性服务业，服务业占地区生产总值的比重达到40%以上，其中文化产业增加值占地区生产总值的比重达到10%以上。

（三）上党城镇群建设创造新范式，打造全省一流城镇群。按照“一核双圈”的城镇空间布局，加快上党城镇群建设，形成中心城区为核心，周边6个县市为内圈，其他5个县为外圈的城镇化体系。搞好旧城改造和新区建设，用三到五年时间，把上党新区打造成高端产业、特色文化、现代生活、生态宜居的现代化新城。建设一批10万人以上大县城、万人以上大集镇和3000人以上中心村，确保城镇化率每年增长2至3个百分点，2016年达到60%以上。积极推进城乡一体化发展，扩大城市公共优质服务资源的辐射带动能力，优化城乡资源配置，努力缩小城乡差别，在产业发展、社会事业、生态建设、社会保障等方面实现城乡统一规划、同步发展、资源共享。

（四）社会文明展现新面貌，和谐长治建设迈出坚实步伐。全民思想道德素质和科学文化素质普遍提升，人民群众权益和社会公平正义得到切实保障，城乡人民生活质量和幸福感明显提高。大力实施科教兴市、文化强市和人才强市战略，加大对科技、教育、卫生等各项社会事业的投入。全市财政性教育经费支出占到地区生产总值的4%以上，全社会研发经费占地区生产总值的比重由1.2%提高到2.4%以上。进一步改善生态环境，森林覆盖率达到40%以上，建成国家生态园林城市、国家森林城市、国家环保模范城市。不断创新安全生产监管机制，全面完成全国第一个本质安全型城市的创建任务。

（五）先行先试闯出新局面，率先走出资源型地区转型跨越发展新路。用足用活综改试验区这个大品牌、大载体、大平台，大胆探索、敢行敢试、会行会试。以更宽的视野和博大的胸怀进一步扩大对外开放，强化与国内外大企业、大集团、大院校的合作，全面提升对外开放水平。不断深化改革，在经济管理、行政管理、社会管理等重点领域和关键环节的改革上取得重大突破，建立起充满活力、有利于创新发展的体制机制，为转型跨越发展奠定坚实的基础。

各位代表，未来五年我们要把长治建成全国一流宜居城市、中部地区经济强市、文化强市、旅游大市和重要的物流基地。只要我们鼓足干劲不放松，狠抓落实不懈怠，就一定能够把宏伟蓝图变成壮丽现实！

三、2012年重点工作

2012年是新一届政府的开局之年。今年政府工作的总体要求是：认真落实省、市十次党代会和全省经济工作会议精神，保增长、保发展、保民生、保稳定，在推动长治快发展、大发展上有新作为，在统筹城乡发展、加快上党城镇群建设上有新进展，在保障和改善民生上有新举措，在社会管理创新上有新成效，保持全市经济平稳较快发展、社会和谐稳定、安全生产形势持续好转，加快建设全国最宜居最宜发展城市步伐，以优异成绩迎接党的十八大胜利召开。

今年经济社会发展的主要目标是：地区生产总值增长15%左右；固定资产投资增长25%；社会消费品零售总额增长17%；进出口总额增长10%；财政总收入和一般预算收入分别增长16%、15%；城镇居民人均可支配收入和农民人均纯收入分别增长12%以上和15%以上；新增城镇就业岗位3.5万个，城镇登记失业率控制在4.2%以内；居民消费价格总水平涨幅控制在4%左右。万元生产总值综合能耗下降3.5%；万元生产总值二氧化碳排放量下降3.7%；万元工业增加值用水量下降3.5%；二氧化硫、化学需氧量、氨氮排放量分别下降2%、1.3%、1%；氮氧化物排放量与上年持平；烟尘、粉尘排放量均下降3%。

围绕上述总体要求和主要目标，今年要全力打好保增长、项目落地、现代农业发展、上党城镇群建设、文化产业发展五大攻坚战，深入推进教育优先、全民健康、创业就业、住房安居、社保扩面五大惠民工程，切实强化人才、科技、金融三大支撑，全面加强安全生产、体制机制、民主法制三大保障，扎实搞好政府自身建设，努力开创经济社会发展新局面。

（一）打好保增长攻坚战，着力保持经济平稳较快发展。

进一步扩大投资。把投资作为拉动我市经济增长的第一动力。今年实施各类重点项目987项，总投资4246亿元，年内完成投资1012亿元。其中续建项目386项，完成投资488亿元；新建项目601项，完成投资524亿元。统筹抓好各个领域的投资，在基础设施建设方面，重点实施中南部铁路大通道、长安高速公路、长治机场改造等97个项目，完成投资69亿元；在产业开发方面，重点实施光电子、多晶硅、现代煤化工等618个项目，完成投资649亿元；在社会事业发展和民生改善方面，重点实施教育、文化、卫生、保障性住房等领域的128个项目，完成投资47亿元；在推进城镇化方面，重点实施企业总部、科教园区、行政中心等135个项目，完成投资121亿元。同时，要继续加大“三农”、科技创新、资源节约等领域的投资。

千方百计帮扶企业保增长。出台帮扶企业保增长、扶持中小微企业健康发展和金融服务实体经济的实施意见。切实加强对宏观经济的监测监控、分析研判，帮助企业搞好市场营销，积极推进企业联合经销抵御市场风险。扎实做好企业重要生产资料的保障，鼓励企业技术创新，开展清理企业收费专项行动，切实解决企业生产、销售过程中的实际困难。巩固煤炭资源整合重组成果，生产矿井要在确保安全的前提下开足马力搞好生产，大力支持煤炭企业发展非煤产业，今年非煤产业产值占煤炭行业总产值的比例达到25%。

推进大中小微企业共同发展。培育一批规模大、效益好、竞争力强的旗舰企业，形成6家年销售收入500亿元以上、10家200亿元以上、15家100亿元以上的三大企业方阵，力争更多企业进入全省“双千亿”、“双五百亿”、“双百亿”行列。加大技术创新、结构调整、人才培养等方面的支持力度，壮大一批发展潜力大、市场前景好的中型企业，新增13个销售收入超亿元的“小巨人”企业。放宽登记条件，免收注册登记费，认真落实税收优惠政策，扶持一批能带动就业、促进增收的小微企业，今年要创办小微企业1000户以上。

（二）打好项目落地攻坚战，着力积蓄经济发展的强大力量。

进一步加快项目建设。切实抓好大项目、好项目建设，以项目结构优化带动产业结构升级。加大传统产业技改项目建设，大力发展循环经济，抓好节能降耗，严格落实项目节能评估制度，严禁新上“两高一资”项目。加快65座保留矿井改造步伐，未开工的要全部开工。推进首钢长钢扩能改造、潞宝6.25米大型捣固焦炉项目建设。电力产业力争新增100万千瓦的装机容量。加快新兴产业项目建设，以20个销售收入百亿元以上的非煤非电项目为重点，顶层设计、高端对接、园区承载、产业运作。快速推进长治光电子工业园区建设，组建国家级的光电子工程研究院（中心）。积极推进潞安1GW太阳能二期、飞利浦照明、黎城多晶硅、100亿安时电力储能电池、潞宝兴海有机合成材料、晨洋太阳能电池、日盛达500兆瓦光伏玻璃、易通环能钕铁硼及微型电机、上泓能源100万吨HF燃油乳化、纳米碳增效肥二期、成功30万辆微车、苏州皇家木屋制造等项目建设，开工180万吨煤基多联产、地下原位煤制气、西门子电机、轻型飞机制造、非晶纳米材料、煤矿乏风氧化利用及设备制造等项目，努力形成新兴产业的集群效应。抓好现代服务业项目，统筹发展会展经济、电子商务、家政服务、社区养老等各类服务业项目，重点推进浪潮长治“云计算”中心、长治物流中心、太行山农产品物流园等项目建设，确保今年服务业增加值增长16%。

进一步加快项目落地。按照项目储备、签约、落地、建设“四位一体”的要求，以中博会、能博会签约项目为重点，加大协调跟进力度，继续实施一个项目、一位领导、一套班子、一抓到底的抓办项目机制，创新项目服务办法，强化项目管理，加快落实土地、资金、环评、能评、水评等手续，加强对投资强度、投资效益的评价考核，把项目落地率作为一票否决的指标，确保我市项目落地资金完成1400亿元。

进一步加强招商引资。按照“高、宽、长、省、园、效”的要求，重点在新兴产业领域引进一批纵向有关联、横向有市场、适合在长治发展、能支撑我市转型跨越发展的大项目、好项目。今年每个县市区的招商引资签约项目不少于15个，落地不少于10个，高新区招商引资增速不低于30%，县市区委书记、县市区长要继续引进一个投资亿元以上的项目，全市签约项目投资总量不少于3000亿元。

各位代表，项目落地是项目建设的生死结。我们要以非常之策、非常之力，扎实做好“项目落地年”的各项工作，以项目建设新成效推动转型跨越大发展！

（三）打好现代农业发展攻坚战，着力促进农业稳定发展和农民持续增收。

加快发展高效特色农业。大力推进六大高效特色产业板块建设，重点抓好300个“一村一品”重点推进村、50个国家级农业标准化示范区、100个现代农业科技示范园、4个省级示范基地县建设。加快推进“一村一品一商标、一县一业一品牌”工程，整体包装推介上党特色农业品牌。启动实施100个特色农产品产业支撑项目，培育壮大沁州黄、达利食品、紫团菌业、鑫四海养殖等30个销售收入超亿元的农业产业化骨干龙头企业，今年农产品加工转化率达到55%。

多种渠道增加农民收入。继续实施“五大增收工程”，新发展10万亩设施蔬菜、20万亩干果经济林、100个规模养殖场、500个农民专业合作社，转移农村劳动力3万人，努力提高农民经营性收入和工资性收入。鼓励农民创业、置业、投资、理财，认真落实各项强农惠农富农政策，促进农民财产性和转移性收入持续增加，确保农民人均纯收入增幅高于城镇居民收入增幅。

不断夯实农业生产基础。加强农业科技创新，健全基层农技推广服务体系和农产品质量安全监管体系。实施百万农民科技大培训、八大农村技能服务人才培养和三百大学生支农计划，建立优质玉米、设施蔬菜等10个现代农业产业技术体系。实行最严格的耕地保护制度，切实加强农田水利基础设施建设，加快推进辛安泉供水改扩建和沁源永和、屯留屯谷等水利工程建设，提高农业综合生产能力，确保粮食总产量稳定在14亿公斤左右。

深入推进新农村建设。抓好240个中心村和长治县振兴新区、壶关常平等11个新农村连片示范区建设，引入城市社区物业公司管理模式，为农民提供水电路气、卫生、通信等公共服务。全面完成省里第二轮“五个全覆盖”工程，同步实施我市新确定的“五个全覆盖”工程。

加大扶贫开发力度。按照新的国家扶贫标准，全面做好扶贫开发工作，切实加大扶贫资金投入。实施产业扶贫、科技扶贫和智力扶贫等综合措施，抓好沁县设施蔬菜、平顺花椒芽菜两大扶贫片区开发，实施60个规模特色产业整村推进项目。深入开展领导干部下乡住村包村增收活动，完善富县帮穷县、大企业扶弱县的工作机制，让扶贫对象更多地共享改革发展成果。

（四）打好上党城镇群建设攻坚战，着力推进城乡一体化发展。

抓好中心城区建设。以新区建设带动旧城改造，以旧城改造提升城市形象，形成新区、旧城共同发展的格局。在新区建设方面，加快以漳泽湖为中心的上党新区建设，大力推进“六大片区”和“十大标志性工程”，完善新区道路管网、园林绿化等基础设施。在旧城改造方面，加快推进“九纵九横”主干道、47条支干道微循环畅通工程，开工世行贷款城市交通项目。对太行街、英雄路等重点路段进行外立面改造，“三河一渠”环城生态水系综合治理工程确保提前一年完成改造任务。所有城中村、城边村、企边村改造全部启动。建设“智慧长治”，全面提高城市管理水平。大力推进建筑节能示范市建设，推广“六个微”绿色环保建筑，完成50万平方米既有建筑节能改造。

加快大县城建设。实施大县城扩容提质战略，膨胀城镇人口。上党城镇群“一核双圈”内圈6个大县城，要以6条城市快速连接线为牵引，在沿线规划建设1至2个各具特色的产业园区，着力构建人流、物流、信息流、资金流、文化流大通道、新兴产业集聚带和人群集中居住带，力争把长治县、襄垣县建成10万人以上的大县城。外圈5个大县城，要完善县城基础设施，提高县城承载力，主动对接中心城市的产业转移，以产业带动人口资源集聚，成为吸纳农村人口就近转移的主渠道。

搞好大集镇、中心村建设。首批确定的53个大集镇要大力发展特色产业，不断完善基础设施，提升公共服务、综合承载和集聚辐射能力。长治县苏店镇、襄垣县下良镇两个省级百强示范镇要在人口集聚、产业发展、公用设施建设等方面取得突破性进展。按照企业带动型、工矿搬迁型、资源交换型、商贸带动型、塌陷治理型、扶贫搬迁型、旅游带动型七大模式，重点建设240个中心村，推进农村集聚化发展、社区化服务。

完善城乡统筹发展新机制。推进上党城镇群在交通体系、水资源综合利用、市政公用设施建设、城乡公共服务和生态建设等方面一体化发展。设立城镇化专项资金，建立城市建设投资公司，鼓励引导煤炭企业和大型企业集团参与城镇化建设。建立以稳定居所、稳定职业、稳定经济来源为条件的落户制度，进一步完善就业、养老、上学、医疗等各项配套政策，积极推进农民进城“六变”、“五有”工作，使新进城人员与原住居民享有同等待遇。扎实做好失地农民的社会保障工作，让农民无论进城还是留乡都能安居乐业。今年全市要新增城镇人口8万人，城镇化率再提高2个百分点，达到50%左右。

深入推进城乡生态建设。围绕创建国家生态园林城市、国家森林城市、国家环保模范城市的目标，加快推进荒山绿化，提升城市绿化品质，完成营造林50万亩，森林覆盖率再提高2个百分点。开展漳泽水库清淤综合治理，打造水秀景美的城中湖。大力实施绿色生态工程，加强对辛安泉水域和上党盆地、长治湿地的保护，逐步关闭淘汰湿地范围内的污染企业。加强大气污染物监测预警，扩展监测项目，逐步将细颗粒物( PM2.5)纳入常规监测体系。加大供热锅炉改造力度，扩大集中供热范围。开工建设污水处理厂二期工程，加快污水处理厂提标升级步伐，推进县城污水处理厂管网配套，确保污水处理率达到95%以上。改造垃圾处理厂，不断完善村收集、镇转运、县处理的垃圾无害化处理网络。搞好重点区域环境综合整治，深化工业点源治理，控制农业面源污染。

各位代表，我市已步入城镇化发展加速期。我们要全力做好上党城镇群建设这篇大文章，走出一条具有中国特色的城乡一体化发展道路，把上党城镇群打造成太行山上的璀璨明珠！

（五）打好文化产业发展攻坚战，着力推进上党文化大发展大繁荣。

大力构建公共文化服务体系。继续实施文化低保工程，开展文化下乡活动，统筹规划建设城市社区活动中心、社区书屋和乡镇文化站、村级文化室、农家书屋等基层公共文化服务设施。大力发展新闻出版、广播影视和文化艺术事业，丰富人民群众的精神生活。加快建设中国古建筑文化园和壁头遗址公园，启动浊漳河谷地古建筑申报世界文化遗产工作。

倾力发展文化产业。树立先进文化观和大文化观，构建具有长治特色的创意设计、动漫游戏、数字视听、演艺娱乐、影视制作、新媒体、移动信息等文化产业体系，提升上党文化的竞争力。加快以长治文化创意传媒产业园为核心的文化“创意谷”建设。筹备建设全球家庭文化永久会址，向商务部申报成立长治国际文化合作（保税）区，成立文化产业投资股份公司。推进文化与旅游、体育、科技的深度融合和集群发展，成立旅游产业发展有限公司，按照统一规划、统一管理、统一开发、统一品牌、统一经营的“五统一”管理模式，加快以太行山大峡谷为龙头的旅游资源及产业整合，做好莱茵湖郡、首席养生特区建设等开发项目，形成具有长治特色的文化旅游产品。加大八路军“两园一剧”宣传推介力度。做大做优中国·长治国际攀岩节等节庆活动。今年文化旅游产业增加值占地区生产总值的比重达到6%。

全力推进文化体制改革。加快经营性文化单位转企改制，组建长治报业、广电传媒和演艺三大集团。推进公益性文化事业单位改革，创新公共文化设施运行机制和管理体制，促进文化资源和要素的合理配置。

（六）深入推进五大惠民工程，着力提高长治人民的幸福感。

实施教育优先发展工程。进一步加大教育投入，确保全市教育支出占一般预算支出的比例不低于18. 34%。大力发展学前教育，今年再新建改扩建89所幼儿园。积极推进义务教育学校均衡发展，搞好薄弱学校标准化建设，每个县要新建或改建一所高标准中学。巩固落实好农村寄宿制小学生“一颗鸡蛋”营养餐工程。全力推进科教园区建设，加快长治职业技术学院与晋东南会校等4所学校的重组，整合师范教育资源组建长治师范高等专科学校。

实施全民健康工程。全面深化医药卫生体制改革，扎实推进县域医药卫生一体化综合改革，完善基本医疗保障制度和基本药物制度，加强全科医生培养和乡村医生队伍建设。加大城乡基层公共卫生服务体系建设力度，稳步推进基本公共卫生服务均等化。巩固城镇居民医疗保险和新农合参保率，政府补助标准在去年的基础上每人再提高40元，达到240元以上。

实施创业就业工程。深入开展创业型城市创建活动，开辟创业“绿色通道”，进一步完善创业政策和服务体系，采取加强培训、减免税费、提供小额贷款等措施，扶持更多有创业愿望和创业能力的人自主创业，以创业带动就业。实施更加积极的就业政策，全面做好农民工、大中专毕业生、城镇就业困难群体和复转军人等重点群体的就业工作。

实施住房安居工程。加快廉租房、公共租赁房、经济适用房、限价商品房建设和农村危房、城市棚户区改造步伐，创新融资渠道，对保障性住房土地供应实行计划单列。今年再开工各类保障性住房1.9万套、169万平方米。全面加强工程质量监管，切实把保障性住房建设成为优质安全的民心工程、德政工程。抓紧完善保障性住房建设、分配、管理、退出机制，探索建立保障性住房分配与赡养老人、计划生育等条件相结合的办法。强化房地产市场监管，巩固调控成果，抑制房价过快上涨。

实施社保扩面工程。进一步扩大社会保险覆盖面，提高统筹层次和保障水平。以非公经济组织、农民工、灵活就业人员为重点，积极开展社会保险扩面征缴专项行动。推进社会保障信息网络建设，年内发放77万张社会保障卡，基本实现参保人员人人持卡。健全城乡最低生活保障动态管理机制，切实做到应保尽保、真正保低。

各位代表，改善民生、提高福祉是政府义不容辞的责任。我们要切实把群众关心、关注、期盼的事情办好办实，让长治人民过上更加殷实幸福的生活！

（七）切实强化人才、科技、金融“三大支撑”，着力增强发展的动力活力。

大力推进人才强市战略。全面贯彻实施中长期人才发展规划纲要，加大人才引进培养力度。加快高层次人才创业园、留学生创业园、企业孵化器、科技创新园等载体建设，筹建山西大学长治创新研究院，今年力争再建8个院士、博士工作站，引进100名海内外高层次人才，培养1万名本土实用型人才，构筑区域人才高地。建立完善人才评价激励机制，努力营造有利于优秀人才脱颖而出、施展才华的发展环境。

大力推进科技创新。加大科技投入，全社会研发经费占地区生产总值比重达到1.4%以上，构建以企业为主体、市场为导向、产学研相结合的科技成果转化和推广平台。加快培育科技创新型企业，新建3家国家级技术研发中心、10家省级技术研发中心，培育2家省级创新型企业。围绕我市20个百亿元非煤非电项目，建立产业技术创新联盟，对关键技术、共性技术组织联合攻关。

大力推进金融创新。积极申报山西金融改革试验区，争取在改革金融机构、培育资本市场、创新融资方式、优化金融生态环境等方面取得突破。引进战略合作伙伴，加快长治商业银行改制步伐，组建长治银行并打造成全省的知名银行。鼓励新型金融组织创新发展，争取民生、兴业等股份制银行入驻长治，新发展10户小额贷款公司，再组建1家村镇银行。建立健全企业信用担保体系，加大金融对实体经济的支持，创新金融产品和服务，不断扩大信贷规模。今年金融机构各项贷款增速要高于地区生产总值增速10个百分点，表外业务融资不低于35亿元。建立多样化的城镇建设投融资机制，发行城镇投资建设债券、市政建设债券，有效拓宽融资渠道。积极推动企业上市直接融资。健全完善农村金融服务体系，组建农业现代化投资公司，加大对“三农”的支持力度。

（八）全面加强安全生产保障，着力推动安全生产形势持续稳定好转。

不折不扣落实“两个主体责任”。把安全生产作为一切工作的底线和保障，严格执行安全生产“双十条”。全面落实政府安全监管责任，各县市区委书记、县市区长对安全生产负第一责任，常务副县市区长对安全生产负全面责任。加强对安全助理、煤矿“八长”、非煤矿山“六大员”、安全监察“五五包保人员”的考核监管。全面落实企业安全生产主体责任，企业的实际控制人必须是企业的法定代表人，对安全生产负第一责任。

不折不扣执行各项安全制度。认真落实省政府安全生产12个行业118条规定，严格执行我市出台的煤矿“5173”、安全生产月例会、部门包保执法、舆论监督、举报奖励等制度。完善安全隐患事前责任追究制度，有效解决思想麻痹、监管软化、玩忽职守、胡干蛮干、制度不落实等问题，在安全生产上必须保证政令畅通、令行禁止，对有章不循、有令不行、有禁不止的现象严查重处。

不折不扣抓好安全生产专项整治。全力推进本质安全型城市创建，深入开展煤矿、非煤矿山、危险化学品、尾矿库、道路交通、森林防火、特种设备、建筑施工、食品药品、中小学校等重点行业领域的安全整治。严厉打击非法违法生产建设行为，加强人防物防技防联动力度，形成“打非治违”的强大合力。创新事故隐患排查机制，突出抓好煤矿瓦斯、水患、顶板隐患治理，对安全生产隐患即报即处，切实做到隐患整改“五到位”、事故处理“四不放过”。

各位代表，安全责任大于天。领导干部保安全，政治生命才安全；企业法人保安全，个人财产才安全。我们要时刻绷紧安全生产这根弦，警钟长鸣、常抓不懈，坚决杜绝重特大事故发生，全力保障人民群众的生命财产安全！

（九）全面加强体制机制保障，着力营造转型跨越发展新优势。

扎实推进综改试验区建设。认真实施综改试验区建设年度行动方案，在重大转型项目、政策创新、板块突破、破解难题四大领域开展先行先试。抓好“一市两县”、“一市两园”、“一县一企”和18个重大标杆项目试点，力争把上党新区建设列入省级标杆项目。在项目用地上用足用活城乡建设用地增减挂钩、矿业存量土地整合利用等政策，创新12项造地办法和6项土地管理机制，探索建立土地“地票”、“飞地”制度。盘活城市土地资源，今年城市土地收储回笼资金不少于20亿元。在资源配置上解决好重大转型项目资源配置问题，内资、外资同等待遇。在节能减排上建立污染排放交易制度，开展碳汇交易，推行阶梯、差别水电价。在行政审批上建立“一个窗口受理、一个科室审核、一个领导审批、一个公章办结”的政务服务模式，按照“环节不减、时限缩短”的原则，加快审批速度。在产业转型上理顺煤炭等资源开发利用体制，建立资源型产业与非资源型产业均衡发展机制，引导资源开发收益主要用于发展接替产业、高新技术产业和服务业。

扎实推进重点领域改革。深化行政、事业单位体制改革，形成高效、快捷、便民的管理体系。扩大集体林权制度改革成果，做好农民承包地、林地、宅基地“三地”确权工作。积极推进市属国有企业股份制、公司制改革，引进战略投资者，与国内外大型企业集团实行优化重组，盘活国有资产。长治县、襄垣县力争进入全国百强县行列。认真落实引导民间投资健康发展的政策措施，鼓励民间资本投资新兴产业和社会公益事业，参与公路、水利等基础设施和市政公用设施建设。对承担重大转型项目和社会公益事业的民营企业，在资源配置、资金保障、土地供应等方面给予重点支持。

扎实推进对外开放。巩固面向德国北威州玛尔市等友好城市合作，不断延伸拓展面向欧美国家的国际经济合作。加强与中原经济区、环渤海地区的交流合作，继续做好中博会、能博会和首届世界晋商大会的参展工作。加快长治海关建设。转变外贸发展方式，强化出口信贷支持，努力扩大兔肉、核桃、风力发电机、汽车、硝酸胺等优势产品出口规模，积极建设光伏光电、建材、镁合金压铸件等外贸转型升级示范基地。

各位代表，体制机制创新是长治发展的强力引擎。我们要用好综改试验区建设这个黄金招牌，不等不靠，主动作为，干成几件先行先试的大事，推动我市在转型跨越的征程中率先突破、勇往前行！

（十）全面加强民主法制保障，着力创造和谐稳定的社会环境。

加强民主法制建设。认真执行人大及其常委会的决议、决定，依法向人大及其常委会报告工作，自觉接受政协的民主监督，广泛听取各民主党派、工商联、无党派人士的意见，提高人大代表建议和政协委员提案办理质量。支持人民法院、检察院依法独立公正行使职权，充分发挥工会、共青团、妇联等群团组织的作用。完善基层民主管理制度，深入推进政务公开、村务公开、厂务公开。加强和改进政府法制工作，做好法律服务和法律援助，深入推进“六五”普法，提高全民法制意识。

加强精神文明建设。大力弘扬太行精神、纪兰精神，丰富发展“平和睿智、包容大度、勤奋敬业、长治久安”的长治人文精神。加强社会公德、职业道德、家庭美德、个人品德建设。深入开展群众性精神文明创建活动，破除陈规陋习，弘扬文明新风。扎实做好全国城市文明程度指数测评迎检工作。

加强和创新社会管理。完善基层社会管理服务，推行城市社区网格化管理，农村实行社会管理网、党建网、农廉网三网合一。健全社会稳定风险评估机制和社会矛盾大调解工作体系，切实解决征地拆迁、劳资纠纷等问题。搞好信访工作，畅通民意诉求渠道，发挥好书记、市长公开电话作用。加强社会治安综合治理，严厉打击各种犯罪活动。建立健全社会预警机制和应急救援体系，提高处置突发事件能力。净化市场环境，打击商业欺诈、制假售假、商标侵权等行为，维护消费者权益。强化网络监管，正确引导网络舆情。支持国防和军队建设，扎实做好双拥共建工作，推进军民融合式发展。搞好人口和家庭公共服务，深入开展“好娃娃”幸福家庭创建工作，加强流动人口管理。积极创建全民健身示范市，努力提高竞技体育水平。进一步加强统计、档案、气象、保密、对台、民族宗教、外事侨务、防震减灾、文物保护、人民防空、地方志等工作。关心支持老龄、妇女儿童、残疾人、红十字会、慈善救助等事业。

（十一）扎实搞好政府自身建设，着力增强政府公信力和执行力。

加强学习，提升能力。认真学习现代科学知识，以全新的理念、高远的境界、战略的眼光，准确把握长治市情和发展大势。用好政策机遇，找准赶超路径，创新推进举措，切实提高驾驭复杂局面、领导转型跨越的本领。

依法行政，从严治政。严格依法履职，规范行政行为，落实好行政执法责任制。完善重大事项公众参与、专家论证、风险评估、合法性审查和政府集体决策相结合的制度，提高决策科学化、民主化水平。全面落实政府系统党风廉政建设责任制，建立健全惩治和预防腐败体系。认真开展重点领域渎职侵权问题专项整治，严肃查处各类违法违纪案件。加强审计监督和行政监察，稳步推进财政预算决算公开，扩大政务公开范围。严格控制“三公”支出，把有限的资源和财力真正用到转型跨越、为民惠民上来。

转变作风，狠抓落实。以铁腕作风、雷霆手段，深入开展机关作风整治，狠刹酗酒、赌博、敛财三股歪风，严查吃拿卡要行为。以“踏石有印”、“抓铁留痕”的精神狠抓落实，坚持一切工作具体化、责任化、现场化、操作化，重点工作、重大事项都要明确责任人，定标准、定进度、定时限，跟踪问效，严格奖惩，强化问责。健全部门间的协调配合机制，多办大局做加法小局做减法的事，少办小局做加法大局做减法的事，形成推动落实的强大合力，切实解决好事办不好、好事办不快、好事办不了的问题。

各位代表，面对光荣艰巨的任务，展望美好灿烂的前景，我们豪情万丈、信心百倍。让我们在省委、省政府和市委的坚强领导下，凝聚全市人民的智慧和力量，为推动转型跨越发展、加快建设全国最宜居最宜发展城市而努力奋斗，以优异成绩迎接党的十八大胜利召开！
